# On Inequality of the Pupils and Iridoplegia

**Published:** 1906-03-17

**Authors:** C. O. Hawthorne

**Affiliations:** Physician to the Central London Ophthalmic Hospital, Assistant Physician to the North-West London Hospital and to the Royal Waterloo Hospital for Children and Women


### Hospital Clinics.

### ON INEQUALITY OF THE PUPILS AND IRIDOPLEGIA.

By C. O. Hawthorne, M.D., M.R.C.P., Physician to the Central London Ophthalmic Hospital, Assistant Physician to the North-West London Hospital and to the Royal Waterloo Hospital for Children and Women.

When a severely critical comparison is made between opposite sides of the body it will frequently be found that there are defects in symmetry more marked than would have been suspected by an ordinary or conventional observation. Occasionally these defects reach a high grade, and then attract more or less general attention. These more gross contrasts may be worthy to be classed as developmental errors, but the slighter and more common degrees of asymmetry exist, not only without appreciable utilitarian or æsthetic prejudice to their possessors, but also apart from the suspicion of any definitely pathological causation. If these statements are true—and they can readily be verified—in reference to the formation of the trunk and limbs, the shape of the head, and the contour of the opposite sides of the face, it is not a matter for surprise to find that they apply in equal measure to the size of the pupils. On the exact percentage of those in whom a distinct difference in this respect can be recognised various observers have come to different conclusions, but all agree that in a certain number of persons free from all evidence of disease one pupil is larger than the other. The numbers have been placed as low as 1 per cent. on the one hand, and as high as 10 per cent. on the other; the sexes appear to be affected in equal degree; and it is generally allowed that the left pupil in these cases of inequality is commonly the larger of the two. In some instances the inequality has been noted in association with difference in the colour of the two irides, and it has been suggested that a less pigmented iris will permit the penetration through its substance of more light than will be allowed by one containing more pigment, and thus the corresponding pupil will receive a stronger reflex stimulus and will therefore be the smaller of the two. Such a suggestion is hardly in accord with the known facts of anatomy and physiology. For the arrangement of the visual nerve tracts is such that light falling on either retina reaches and stimulates the reflex pupillary centre for each eye, and hence, even though the amount of light admitted to the two eyes may differ, the total stimulating force acting on each reflex centre is approximately the same. Further, and as a matter of fact, difference in the size of the two pupils, in the circumstances now under discussion, may exist apart from differences in the tint of the irides, and, conversely, the irides may be differently pigmented, and yet the pupils be equal in size. Upon what element or factor these peculiarities depend can at present be only a matter of conjecture, and it is not impossible that the exact localisation of the minute flaws in the process of development responsible for them, and for other minor errors of symmetry, may long or even permanently defy embryological analysis. But to the physician, though the question of etiology may here make little appeal, the fact itself that occasionally a person may be found in whom, and merely as a personal peculiarity, the pupils are unequal, is of great moment. For it is a matter of common clinical experience that such a condition may be a piece of evidence of dominating importance in an individual diagnosis and prognosis. Hence, when difference in the size of the pupils is discovered, it becomes necessary to recognise that the fact may or may not have a serious significance. And the selection of the alternative must largely be determined by the answer to the question, Is the inequality a new development? To secure a confident answer to this question is, however, not always an easy task. Persons whose pupils have never been equal may live for years without discovering the fact until some symptoms of ill-health or other accidental circumstance causes a more or less critical self-examination. Hence, the condition may be presented as a new development when it is really of very old standing. One practical lesson that arises from these considerations is that it would be well were the pupils more commonly, and as a matter of routine, included within the scope of the ordinary clinical examination. The information obtained in this way is often of the highest significance, and is, whether in its positive or negative aspects, at least as valuable, and certainly not less precise, than that afforded by the time-honoured examination of the pulse and inspection of the tongue. One result, were the practice now advocated commonly adopted, would be that what may be called inequality of the pupils natural to the patient would usually be discovered in early life, and, provided the fact were made known to the patient or his friends, the risk of an anxious interpretation being applied to it in later life would be avoided. The theory or view that ocular conditions or symptoms are mainly the concern of the ophthalmic surgeon is far too generally adopted. They have, on the contrary, the closest possible relationship to the work of the physician and the family practitioner, and ought to be made the subject of routine observation.

The question may now be asked whether inequality of the pupils may occur as the first event in the evolution of any form of organic disease. It is, of course, well known that such a condition is an important and not infrequent symptom in such diseases as tabes dorsalis and general paralysis of the insane. But here there are usually other defects such as bilateral loss of the light response, in addition to various evidences in other parts of the body, not to speak of mental abnormalities. And certainly, apart from such clinical facts, mere inequality of the pupils would be but a slender foundation for the diagnosis of either of the serious diseases just named. The point now to be dealt with is rather that presented by a conspicuous difference in the size of the pupils regarded as a new fact, and in a person otherwise free from other evidence of disease. First, as already stated, there is the possibility that what is stated to be new is really the recent discovery of an old fact. Supposing, however, that the larger pupil is not only dilated, but that it fails to react to light when this is applied both to the same and to the opposite eye—that is, both the direct and consensual light response are absent—it is obvious that there is not only a dilatation of the pupil, but also a paralysis of the sphincter iridis. In such circumstances, unless there is positive evidence in support of such a view, it will be difficult to believe that the condition is merely an individual peculiarity. In short, inequality of the pupils with persisting light-response in each is, unless the contrary can be shown, probably a condition natural to the individual. But with a dilated and immobile pupil the presumption is in the opposite direction, and unless such a combination can be proved to have existed from early life, it must be condemned as definitely pathological. With disease of, or injury to, the optic nerve or retina on one side, some disturbance of the corresponding pupil is, of course, to be expected; but here vision will be defective, the ophthalmoscope will show changes in the fundus, and whilst the direct light-response is absent, the pupil will contract when light is allowed to fall on the opposite retina. In the absence of all these conditions the interpretation of a dilated and immobile pupil calls for further inquiry. The first suggestion which arises is that by accident or design the patient has managed to come into contact with some preparation of belladonna or atropine. If this is the case, there will not only be dilatation of the pupil, but also paralysis of the accommodation, and to read small type at a distance of 12 inches or less will be impossible. Further, in the course of ten days or so the paralysis in both respects will, unless the application is renewed, disappear. Supposing, however, that the dilated and immobile pupil can be shown to be a new fact, and, further, that the influence of a mydriatic can be excluded, what diagnostic and prognostic values are to be attached to it? In answer to this it must be recognised that the condition is a definite paralysis, and that its existence therefore throws doubt on the integrity of the nervous system. An extra-ocular paralysis, it will be allowed, has this significance, and it is reasonable to apply the same conclusion to a paralysis of the sphincter iridis. Further, there is distinct clinical evidence that a paralysis of very limited distribution, and sometimes also of limited duration—an iridoplegia, a cycloplegia, a diplopia, for example—may be the first event in the clinical manifestation of serious degenerative nervous disease, and, what here has specially to be emphasised, that such paralysis may long precede any developments of more precise diagnostic significance. It therefore follows that any one of these events—a dilated and immobile pupil, for example—must excite some anxiety, and this more especially when the patient is one who must be regarded as predisposed to such disease on account of syphilitic infection or other cause. On the contrary, when these latter influences can be excluded, whilst there must be some degree of fear in reference to the future both of the individual lesion and the nervous apparatus generally, it is not impossible that an iridoplegia may have a relatively benign explanation. Such indefinite terms as "cold" and "rheumatism" would appear to be responsible for many facial palsies and also for some paralyses of the extra-ocular muscles. Their aid may be invoked in occasional instances of dilated and immobile pupil, though such an explanation must be advanced with mental reservations and qualifications. Altogether, therefore, inequality of the pupils and an iridoplegia are in much the same position as that occupied by other varieties of ocular paralysis. Each may possibly be a congenital peculiarity; each, on the one hand, may signify commencing nervous disease, or may, on the other, be due to some uncertain and comparatively unimportant element which more or less accurately may be described as rheumatic. In addition, the possibility of atropine, cocaine, or other mydriatic must not be forgotten.

The discussion of inequality of the pupils can hardly be closed without a reference to this condition when it results from lesions or disturbances within the thorax. An aneurism is perhaps the best known of these, but tumour, phthisis pulmonalis, pleurisy, and pleural effusion, have also been held responsible for pupillary inequality. The usual explanation offered is that these conditions, one and all, produce the inequality by disturbing the functions of the sympathetic nerve, in the upper dorsal and cervical part of which the fibres supplying the dilator pupillæ are to be found. This explanation certainly does not meet all cases, and perhaps it is safest to fall back on two elementary physiological laws, namely, that all peripheral irritations dilate the pupil, and secondly, that all reflex movements manifest themselves first on the side to which the irritation is applied.

